# Partial cystectomy for bladder squamous cell carcinoma with a 10-year follow-up: a case report

**DOI:** 10.3389/fonc.2023.1237228

**Published:** 2023-08-09

**Authors:** Na Yin, Wei Zhao, Tao He, Tingchao Li, Xu Lei, Hao He, Zongmin Long, Yan Wang

**Affiliations:** ^1^ Department of Urology, The Third Affiliated Hospital of Zunyi Medical University (The First People’s Hospital of Zunyi), Zunyi, China; ^2^ Department of Pathology, The Third Affiliated Hospital of Zunyi Medical University (The First People’s Hospital of Zunyi), Zunyi, China

**Keywords:** partial resection, urinary bladder neoplasms, carcinoma, squamous cell, diagnosis, pathology, prognosis

## Abstract

Squamous cell carcinoma (SCC) of the bladder is a rare malignancy of the urinary system. It is prone to invasion and metastasis in the early stage and has a poor prognosis. This case reports a 65-year-old female patient with SCC of the bladder who was free of disease recurrence and metastasis 10 years after partial cystectomy (PC) combined with left ureteral reimplantation. The treatment plan and admission of this patient were retrospectively analyzed in order to provide some reference significance for the treatment plan for the SCC of the bladder.

## Introduction

Squamous cell carcinoma (SCC) of the bladder is a rare urologic malignancy, with an estimated incidence of 3% to 5% of the bladder (1). Due to its rarity, no consensus or recommendations have been reached on how to manage SCC of the bladder. At this stage, the main treatment methods are radical cystectomy (RC) and total cystectomy. However, these two surgical approaches seriously affect the long-term quality of life of patients because PC does not completely clear the lesion, which is easy to recur and metastasize in the later stage. Therefore, it is rarely used as a surgical option for patients with SCC. Here, we report a 65-year-old female patient with SCC of the bladder who underwent partial cystectomy. The patient did not receive further treatment and did not experience tumor recurrence or metastasis in the past 10 years. The patient did not receive further treatment after surgery, and no signs of tumor recurrence or metastasis were observed at 10 years of surveillance.

## Case report

A 65-year-old woman was admitted to the hospital with a 1-week history of bladder occupancy on physical examination. The woman complained of mild urinary frequency. There was no fever or low back pain. There were no urinary symptoms such as urinary urgency, painful urination, and difficulty in urination. The patient denied a history of smoking, exposure to chemical materials, or residence in an infected area. Specialized examination revealed no percussion pain in bilateral renal areas. There was no erythema or elevation. There was no deep pressure pain in the ureteral travel area bilaterally. The bladder was not filled, and there was no pressure pain. The vulva exhibited no erythema or neoplasia in the external urethral opening. Routine blood tests suggested abnormal neutrophils. Urinalysis shows positive occult blood in addition to red blood cell and leukocyte abnormalities. None of the remaining results showed significant abnormalities. After a urinary tract infection was identified, we collected morning urine bacteriological cultures for drug susceptibility analysis.

B-scan ultrasonography showed a strong echo nodule of approximately 1.8 × 1.7 × 1.1 cm in size on the posterior wall of the bladder. Pelvic computed tomography (CT) scan showed that the bladder tumor had invaded the inner part of the left ureteral wall with a diameter of approximately 12.0 mm. Kidney, ureter, and bladder (KUB) examination showed no abnormality. Intravenous pyelogram (IVP) showed severe hydronephrosis in the left kidney, with full development of the left ureter. After controlling the infection, we performed a cystoscopy. Through cystoscopy, mild elevation of the bladder neck and mild trabecular formation in the bladder were observed. Scattered patchy infiltrative lesions were seen in the posterior wall of the bladder apex with a maximum extent of approximately 3.5 cm × 4.5 cm. A white coral-like mass of approximately 0.8 cm in diameter was seen in the left wall of the bladder. There was a coral-like mass of approximately 2.0 cm in diameter at the opening of the left ureteral orifice, and the left ureteral orifice was not seen. Three tissues were taken and sent for examination, and the pathological examination returned showed grade I uroepithelial cell carcinoma of the bladder. The clinical diagnosis was T3bN0M0 stage of uroepithelial carcinoma of the bladder.

After the relevant investigations were completed, PC combined with left ureteral reimplantation was immediately performed. Under general anesthesia, an incision of approximately 18 cm was made in the middle of the lower abdomen, and a layer-by-layer incision was made until the bladder was cut. No stones or foreign bodies were seen in the bladder. A tumor with an ectophytic growth of approximately 2.0 × 2.0 cm in diameter can be seen at the opening of the left ureter, and the left ureteral opening was not visible. The tumor was excised 2 cm from the edge of the tumor, reaching deep into the muscular layer. The wound was closed with interrupted sutures with 3/0 absorbable thread. The left ureter was found at the iliac vessels, and it was observed that the ureter thickened approximately 1.0 cm in diameter. The left ureter was cut and trimmed by freeing it sufficiently down along the iliac vessels to the bladder wall segment. The bladder was incised at the left base of the bladder for approximately 1 cm. The distal end of the left ureter was introduced into the bladder and was sutured to the bladder mucosa with a 4/0 absorbable suture. A No. 5 double J tube was left in place between the ureter and the bladder. After satisfactory anastomosis, a 4/0 absorbable thread was sutured and fixed outside the bladder. When there was no active bleeding, the incision and bladder were flushed with sterile water. The bladder and the incision were soaked with hydroxycamptothecin 20 mg + saline 50 ml for 10 min. The bladder was then closed with continuous full sutures with 2/0 absorbable thread. A drain tube was placed in the left pelvis after the procedure, and the incision was finally sutured layer by layer.

**Figure 1:**
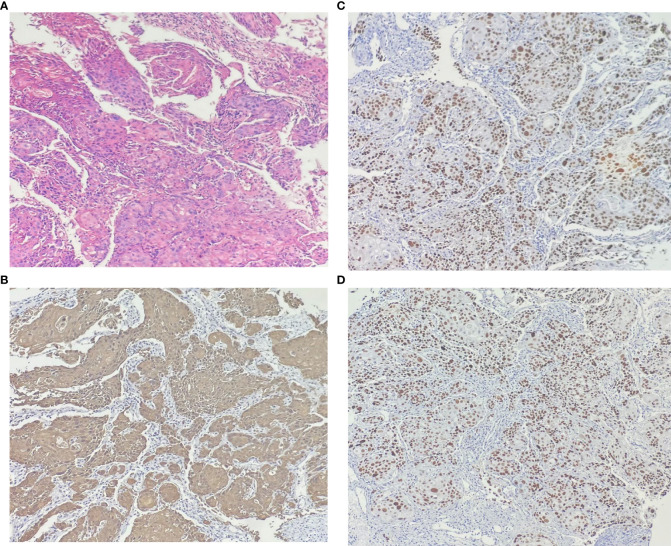
The results of hematoxylin-eosin staining: **(A)** Microscopic pathological nuclear fission image, keratin pearls and intercellular bridges are present in many cases of well-differentiated carcinoma. No components of transitional epithelial carcinoma were seen. Immunohistochemically: the cell membranes and cytoplasm were positive for **(B)** CK5/6. The nucleus were positive for **(C)** p40 and **(D)** p63.

In our case, the woman was diagnosed with SCC of the bladder grade 1 based on pathological staining of the postoperative specimen. H&E staining (**Figure 1**) microscopically showed heterogeneous proliferating squamous epithelial cells with infiltrative growth. Immunohistochemically (IHC), the tumor cells were positive for CK5/6 (**Figure 1B**), P40 (**Figure 1C**), and P63 (**Figure 1D**). The clinical diagnosis of SCC of the bladder at stage T3bN0M0 was made by excluding metastatic lesions from other sites. The patient and her family refused further total cystectomy combined with urethral diversion. Postoperative symptomatic treatment such as anti-infection, hemostasis, and fluid replacement was given.

**Figure 2:**
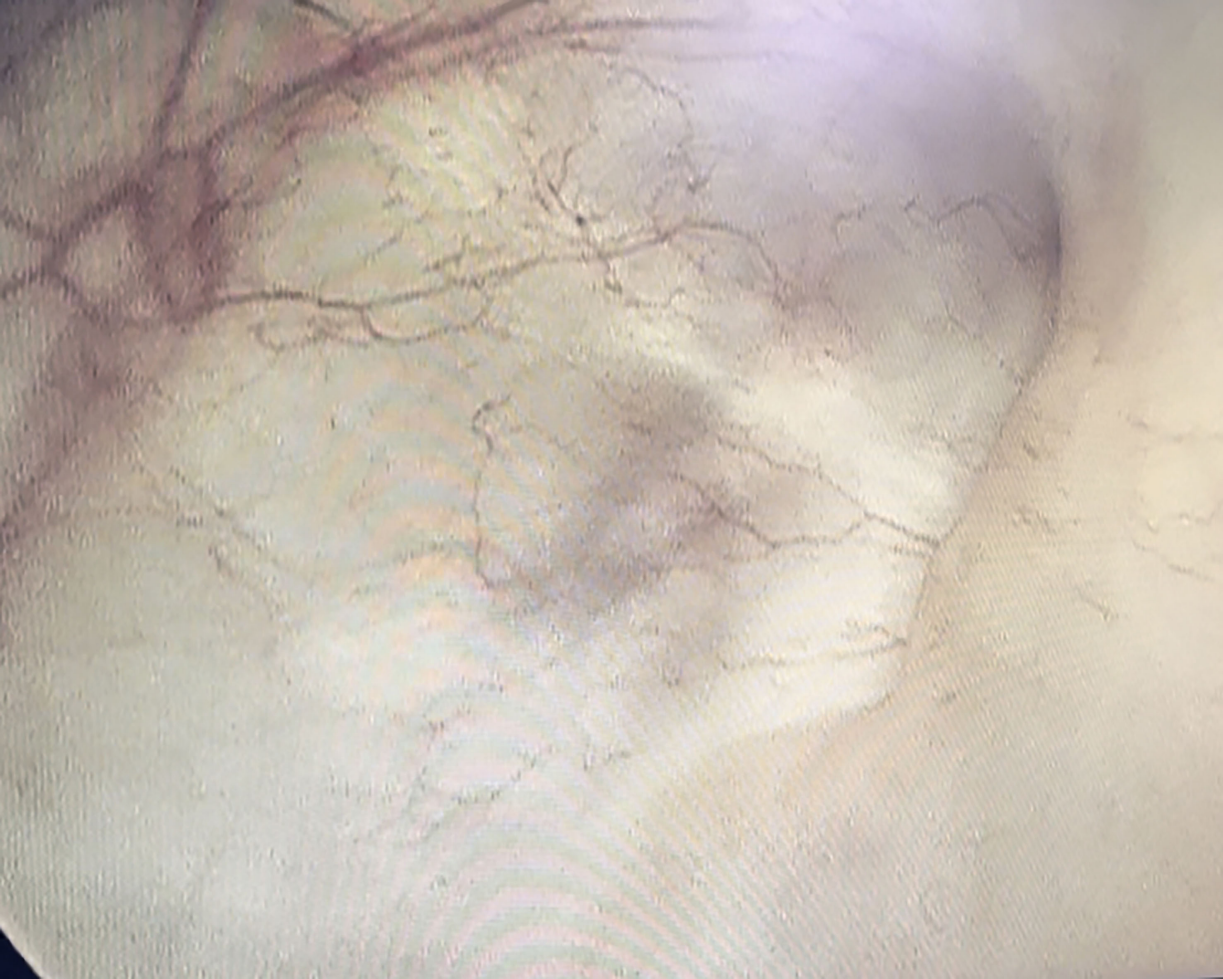
Cystoscopy showed good bladder filling and no new organisms were seen. The left ureteral opening was higher than the right, and scarring changes were seen after surgery.

**Figure 3:**
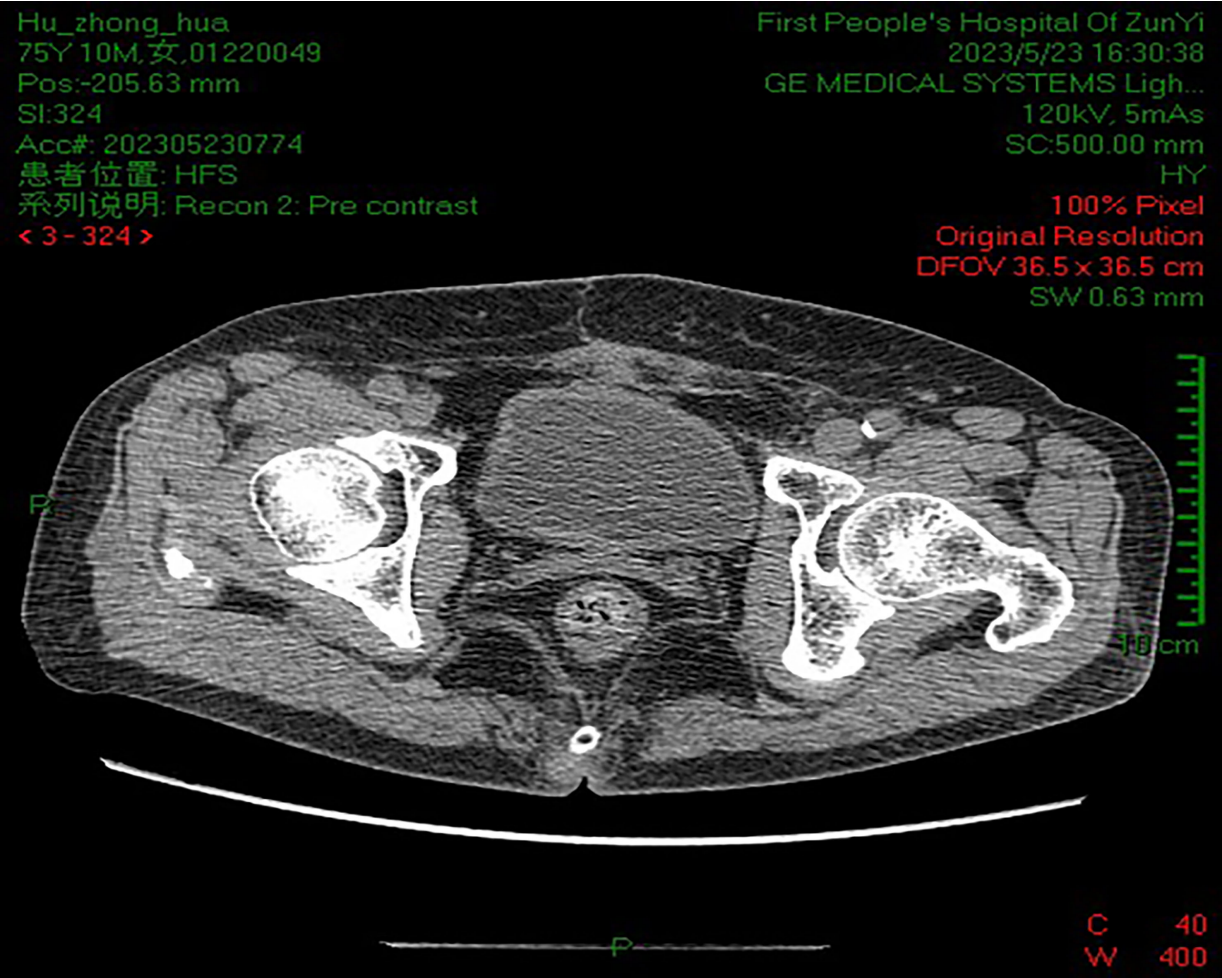
Pelvic CT did not show tumor recurrence and metastasis.

Subsequently, the woman was discharged after 15 days of postoperative improvement of symptoms. After surgery, the woman received regular outpatient chemotherapy with bladder irrigation and follow-up cystoscopy and CT at our clinic. Ten years have passed since then, and the patient’s prognosis is good. A repeat cystoscopy (**Figure 2**) and pelvic CT (**Figure 3**) showed no signs of recurrence or metastasis.

## Discussion

According to the 2020 Global Cancer Statistics, bladder cancer is the 10th most common cancer in the world. More than 200,000 people die from bladder cancer worldwide each year. The disease is more common in men than in women. At this stage, the incidence rate tends to be stable or decreasing in men but is increasing year by year in women (2, 3). Depending on histological sources, 90%–95% of bladder cancers originate from urothelial carcinoma, which is called transitional cell carcinoma. The remaining fraction consists of non-urothelial carcinoma (non-UC). Among them, SCC is the most common histological type of non-UC of the bladder. SCC is more common in women, which may be associated with the predisposition of women to chronic urinary tract infections and cystitis (4, 5). SCC of the bladder is highly malignant, with 5-year overall survival (OS) of approximately 23%. Among them, 33% were non-muscle-invasive SCC, 28% were muscular infiltrates, and 6% were metastatic SCC (6, 7).

It is reported that SCC usually occurs in the lateral wall and triangular area of the bladder. In addition, it may also occupy the diverticula or even extend locally into the urethra or ureters (8). At present, the pathogenesis of SCC of the bladder is still unclear. It is generally considered to be associated with risk factors such as long-term smoking, recurrent urinary tract infections, bladder stones, schistosomiasis infection, mucosal leukoplakia, and long-term indwelling catheter after spinal cord injury. At the same time, these factors can also contribute to each other and continuously stimulate local recurrent chronic inflammation of the bladder mucosal tissue. Further stimulation can mediate squamous metaplasia of the metastatic epithelium, eventually leading to carcinogenesis (9). Therefore, early prevention can be based on these risk factors. This patient has no history of smoking, which excludes SCC due to smoking. Imaging the urinary system can further rule out the influence of stone factors. The absence of a history of schistosomiasis infection further excludes schistosomiasis-associated SCC. The woman had mild urinary frequency on admission. The blood routine showed abnormal neutrophils, and urinalysis indicated abnormal white blood cells as well as red blood cells, which indicated that chronic infection might induce SCC of the bladder.

As a specific type of bladder cancer, there is a lack of specificity in clinical symptoms between the SCC of the bladder and uroepithelial carcinoma (UC). The majority of patients are asymptomatic in the early stages, with hematuria as the main clinical feature in 63%–100% of patients in the late stages and bladder irritation in the other two-thirds (10). Currently, the diagnosis of SCC of the bladder is challenging. B-scan ultrasound is usually used for initial screening. CT and MRI are used to diagnose the depth and extent of the invasion, which can help with staging and the choice of treatment. Urine cytology has strong specificity for detecting high-grade urothelial carcinoma, but data from the study of Soave et al. (11) showed that only a quarter of patients with SCC were positive for circulating tumor cells. Another report showed a sensitivity of only 39% for transurethral resection of bladder tumors (TURBT) (3). It is important to note that multi-site mucosal biopsy can reduce the chance of missed or even misdiagnosis. In this case, only three tissues were taken for biopsy from three lesions under cystoscopy before surgery, and the pathology back showed UC grade I. H&E staining of the pathological tissue after PC showed SCC grade I. Thus, the clinical diagnosis mainly relies on the pathological histological examination of the gross specimen.

SCC of the bladder has a poor prognosis, with most patients dying within 1–3 years of diagnosis (10). There is a lack of randomized prospective data to guide clinical treatment due to the limited number of clinical patients. Surgical treatment for SCC includes total cystectomy, PC, and TURBT. Although neoadjuvant therapy and adjuvant chemoradiotherapy have achieved varying degrees of success in the treatment of bladder cancer, there is no uniform standard for efficacy in SCC. Surgery can cure localized cancer, while palliative chemotherapy or radiation therapy remains the mainstay of treatment for patients with unresectable and metastatic bladder cancer. Compared to UC, non-UC is highly malignant and often has micro-metastases at the time of presentation (12). Resection of local lesions may be more important than radiotherapy. RC is an effective treatment for limited SCC and has shown better survival rates when compared to other treatments such as PC, radiotherapy, and chemotherapy (9). Ehdaie et al. (13) found from the National Cancer Database that the median OS for patients with SCC who received concurrent chemoradiotherapy after TURBT was 15.1 months. In contrast, the 5-year OS of patients with bladder SCC after RC surgery could reach 40%. Early diagnosis as well as treatment is essential to improve the prognosis. The European Association of Urology guidelines classify non-muscle-invasive bladder cancer as a high-risk tumor and recommend RC treatment after early diagnosis (7). For the same reason, the American Urological Association and National Comprehensive Cancer Network guidelines recommend RC treatment for T1 patients with non-UC based on expert opinion (4).

However, SCC usually presents as locally advanced and is often not suitable for PC surgery. Sometimes, it cannot be removed by RC surgery either. More recently, there have not been any major series considering PC (8). Bladder preservation is generally indicated in patients who are older or whose comorbidities are not candidates for RC. A report by Brocklehurst et al. (3) confirmed the excellent efficacy of bladder preservation in small cell carcinoma of non-UC. However, SCC is not supported by good evidence. In another study, bladder-preserving chemoradiotherapy (BPCRT) for muscle-invasive bladder cancer in non-UC had similar overall survival to cystectomy but worse OS for SCC. The median OS for BPCRT for SCC was 12.6 months, with a 5-year OS of 21.7% (14). In another study, Luzzago et al. (15) found that non-UC 5-year cancer-specific mortality (CSM) after PC surgery was higher than in UC patients.

However, given that decreased quality of life and emotional stress are often associated with urethral diversion after total cystectomy, it is generally difficult for patients to accept this procedure. Compared to conventional RC, PC not only maintains the integrity of the urinary system but also avoids the complications of stones and metabolic syndrome that accompany urethral diversion (16). Nevertheless, this procedure also has certain disadvantages, such as the inability to clear micrometastases and the tendency of later tumor recurrence and distant metastases. It has been reported that approximately 90% of patients who undergo treatment die from renal failure caused by tumor recurrence at the urethral anastomosis (17). Therefore, PC is not used as the preferred treatment option in clinical practice. Hydronephrosis, advanced age (70 years or older), lymphovascular invasion, lymph node metastasis, and advanced T-stage have been reported as negative prognostic factors for SCC. Among them, hydronephrosis is an independent risk factor (18). IVP may show hydronephrosis in 33%–59% of cases (18). In this case, IVP showed severe hydronephrosis in the left kidney. The pelvic CT showed that it was due to tumor involvement of the inner segment of the left ureteral wall, which was consistent with the site of SCC involvement reported in the literature.

As the first pathological diagnosis of UC was made in this patient, imaging suggested ureteral invasion. The clinical stage was T3bN0M0. Therefore, we performed PC combined with left ureteral reimplantation. PC not only removed the local lesion but prevented further metastasis as well as recurrence of the tumor. Combining ureteral reimplantation further alleviated the renal damage caused by hydronephrosis. PC postoperative pathology showed grade I SCC. Microscopically, pathological nuclear schizophrenia was seen on H&E staining, and keratin pearls and intercellular bridges were present in many cases of well-differentiated to moderately differentiated carcinoma, with no UC components. IHC showed that CK5/6 was expressed in the cell membrane and cytoplasm; P40 and P63 were expressed in the nucleus, which is consistent with the results of SCC-specific immune indicators (10). In 2021, Deuker et al. (19) found that among all non-UC patients, the TNM stage at diagnosis tended to be higher than in UC patients. Considering that higher-stage SCC exhibited higher CSM, we recommended that the case patient undergo further total cystectomy with urethral diversion, but the patient and her family refused further treatment. After surgery, the patient was instructed to undergo regular bladder irrigation chemotherapy and regular cystoscopy as well as CT follow-up at our outpatient clinic.

In recent years, the patient, in this case, has had a good prognosis. There is no sign of tumor metastasis as well as recurrence. On the one hand, it may be related to early diagnosis. The patient was found to have an occupying bladder lesion on physical examination and was admitted with only mild urinary frequency and no meatus hematuria. On the other hand, SCC patients with T3/4 disease usually exhibit a lower rate of lymph node metastases (10%–25%) and distant metastases (8%–27.7%) (20). This case is consistent with the report of ERDEM, which showed no lymph node invasion and pelvic metastasis on CT 10 years ago. Meanwhile, Luzzago et al. (15) compared CSM of 5 years after RC and PC surgery in non-UC and UC patients and found that the prognosis of non-UC was not related to the mode of resection but rather stemmed from the histological type. Furthermore, while RC is the recommended treatment of choice, other less damaging alternatives such as PC should be considered. Although this patient had a long tumor-free survival after PC surgery, more cases and follow-ups were needed to improve the understanding of PC in patients with SCC of the bladder.

## Conclusion

In summary, SCC of the bladder has a higher recurrence rate and a lower survival time. Early diagnosis and treatment are key to improving prognosis. Individualized treatment plans should be developed according to the stage and grading of the tumor, tumor type, size, presence or absence of metastasis, and the patient’s systemic condition. To our knowledge, this case involves the longest recurrence-free survival time among patients with SCC of the bladder after PC surgery published thus far. This case may provide some reference significance for the surgical approach of patients with SCC of the bladder.

## Data availability statement

The original contributions presented in the study are included in the article/supplementary material. Further inquiries can be directed to the corresponding author.

## Ethics statement

Written informed consent was obtained from the individual(s) for the publication of any potentially identifiable images or data included in this case study.

## Author contributions

NY collated, wrote and revised papers. YW is responsible for proposing research ideas and writing guidance. TH provided the case studies in this article, and conducted preliminary literature screening and result analysis. HH, XL and ZL searched and screened secondary literature; TL provides pathological diagnosis guidance; WZ conceives and designs the article and is responsible for the article as a whole. All authors contributed to the article and approved the submitted version.
